# Improving the representativeness of the tribal behavioral risk factor surveillance system through data integration

**DOI:** 10.1186/s12889-023-15159-z

**Published:** 2023-02-07

**Authors:** Sixia Chen, Janis Campbell, Erin Spain, Alexandra Woodruff, Cuyler Snider

**Affiliations:** 1 Department of Biostatistics and Epidemiology, University of Oklahoma Health Sciences Center, Oklahoma City, OK USA; 2 Southern Plains Tribal Health Board, Oklahoma City, OK USA

**Keywords:** Data integration, Nonprobability sample, Selection bias

## Abstract

**Background:**

Previous literature showed significant health disparities between Native American population and other populations such as Non-Hispanic White. Most existing studies for Native American Health were based on non-probability samples which suffer with selection bias. In this paper, we are the first to evaluate the effectiveness of data integration methods, including calibration and sequential mass imputation, to improve the representativeness of the Tribal Behavioral Risk Factor Surveillance System (TBRFSS) in terms of reducing the biases of the raw estimates.

**Methods:**

We evaluated the benefits of our proposed data integration methods, including calibration and sequential mass imputation, by using the 2019 TBRFSS and the 2018 and 2019 Behavioral Risk Factor Surveillance System (BRFSS). We combined the data from the 2018 and 2019 BRFSS by composite weighting. Demographic variables and general health variables were used as predictors for data integration. The following health-related variables were used for evaluation in terms of biases: Smoking status, Arthritis status, Cardiovascular Disease status, Chronic Obstructive Pulmonary Disease status, Asthma status, Cancer status, Stroke status, Diabetes status, and Health Coverage status.

**Results:**

For most health-related variables, data integration methods showed smaller biases compared with unadjusted TBRFSS estimates. After calibration, the demographic and general health variables benchmarked with those for the BRFSS.

**Conclusion:**

Data integration procedures, including calibration and sequential mass imputation methods, hold promise for improving the representativeness of the TBRFSS.

## Introduction

Prior research revealed significant health and behavioral risk factor disparities between American Indians and Alaskan Natives (AI/AN) and other racial groups. For instance, in 2017–2018, the US age-adjusted prevalence of diabetes for AI/AN adults was 14.7%, compared with 7.5% for non-Hispanic White adults [1]. According to the CDC [2], in 2018 the age-adjusted obesity prevalence for AI adults was 48.1% compared with 30.0% for Non-Hispanic Whites. Additionally, AI/AN youth and adults have the highest prevalence of cigarette smoking among all racial/ethnic groups in the US [3, 4].

However, studying the AI/AN population is challenging due to the small population size, high heterogeneity, geographic spread, and poor representation of the existing survey data. In 2019, AI/AN represented 1.7% of the US population, with 5.7 million AI/AN nationwide [5]. AI/AN populations in many states are very small, ranging from 9,006 in Vermont to 772,394 in California [5]. In 2021, US tribal populations ranged from about 400 for Coachella California to 389,751 for Cherokee Nation in Oklahoma.

Recent COVID-19 studies have included AI/AN with a sample size of only three [6], while others lumped AI/AN with other races [7–9]. Still others ignored the population altogether [10–17], while some considered AI/AN without discussing misclassification [18–20], and others focused solely on AI/AN populations [21–25]. In addition, American Indians and Alaskan Natives are often lumped into the same group, but have different histories and lifestyles [26]. In fact, most tribes in the US have very different histories [27], economic structure (27), political structure (27), health care access [28], social vulnerability [29], levels of overcrowding, historical trauma [30–32] and population mixing [26].

Representative survey data for AI/AN are often limited [33]. A 2013 review of US population surveys from 1960 to 2010 for cancer research found only 17 surveys with AI/AN work, and many of those had less than 500 respondents [34]. Due to the sparseness of AI/AN populations, some surveys, such as National Health Nutrition and Examination Survey (NHANES), do not release identification information for AI/AN. The Oklahoma Tribal Epidemiology Center (OKTEC) Tribal BRFSS (TBRFSS) provides a unique data source for AI/AN with a sample size over 700, providing rich information related to social demographics, health, and behavior. Such information has been used by OKTEC for designing intervention strategy for improving AI/AN health. TBRFSS collected high quality data for AI/AN by using a well-developed sampling design of a combination of event sampling, email sampling, and social media sampling from Kansas, Oklahoma, and Texas. However, it may suffer from selection bias since the sampling design for TBRFSS is non-probability (e.g. not every unit in AI/AN population had non-zero probability of being selected), see Baker et al. (2013) for more discussion about issues with non-probability samples [35].

Data integration procedures, by combining information from probability samples and non-probability samples, can be used effectively to reduce selection bias of non-probability samples (see [36–38], among others). Calibration methods [39, 40] have been used frequently in practice by constructing the calibrated weights in non-probability sample such that the weighted estimates by using a non-probability sample will benchmark with the estimates from the probability sample. The underlying assumption for using calibration methods is that the non-probability sample and the probability sample should have some overlap variables, which will often be satisfied in practice. Mass imputation [38, 41, 42] is another set of data integration methods that borrow the strength of statistical modeling to predict the outcome variables in the probability sample after fitting prediction models by using the non-probability sample. The power of the mass imputation procedure depends on the effectiveness of the modeling process.

In this paper, we propose using calibration methods and mass imputation by fully conditional specification (FCS) [43, 44] to improve the representativeness of the TBRFSS. The imputation by fully conditional specification (FCS) method has been shown to be very effective for handling multivariate missingness in practice [45, 46]. To the best of our knowledge, we are the first to consider such a method in data integration problems. Previous literature did not consider mass imputation with multiple outcome variables. In addition, we are the first to consider using data integration methods to reduce the selection bias for AI/AN populations. Our proposed data integration methods are important for AI/AN research because most samples for AI/AN research are non-probability samples, which may suffer from serious selection bias.

Our paper is organized as follows. In section two, we describe the BRFSS and TBRFSS surveys. We discuss our proposed data integration methods in section three. Results are presented in section four. Final remarks, including future research and limitations, are presented in section five.

## Background for BRFSS and TBRFSS

### 2018 and 2019 Behavioral Risk Factor Surveillance System (BRFSS)

The Behavioral Risk Factor Surveillance System (BRFSS) is the nation’s premier system of health-related telephone surveys that collect state data about U.S. noninstitutionalized adults regarding their health-related risk behaviors, chronic health conditions, and use of preventive services. It provides large scale (over 840,000 completed cases in 2018 and 2019 together) national- and state-level representative samples (e.g., probability samples) that use a stratified sampling design with both landline and cell frames. The sampling is done independently for each state. Together, the 2018 and 2019 BRFSS collected information for over 11,500 adults (with over 970 AI/AN adults) in Oklahoma. For more detailed information about the sampling and weighting of the BRFSS, see https://www.cdc.gov/brfss/annual_data/2019/pdf/overview-2019-508.pdf.

### 2019 tribal Behavioral Risk Factor Surveillance System (TBRFSS)

The goal of the 2019 TBRFSS was to survey American Indian populations in Kansas, Oklahoma, and Texas to determine the extent of behaviors that contribute to or protect from adverse health outcomes. Convenience sampling was used to obtain responses to the survey. AI/AN representation in surveys and sampling is often small and limited. The OKTEC serves as the public health authority for 43 federally recognized tribes in the Southern Plains Area (Kansas, Oklahoma, and Texas), which had an AI/AN population of approximately 443,734 in 2017. The OKTEC and the University of Oklahoma Health Sciences Center Hudson College of Public Health have a longstanding relationship and have previously conducted projects together. The TBRFSS is a deliberate oversampling to have a better understanding of the health factors in American Indian communities. The compiled analyzed data will then go back to the tribes to provide information for making decisions with programs, applying for grants, and to help with other public health outcomes.

The survey collection was conducted in three phases to try to capture a more complete sample of the AI/AN population. Each of the three phases had a drawing for incentives, with three larger awards available to be drawn from the participants within each phase. The first phase was conducted from September to December 2019 with a mix of convenience sampling by attending tribal events in person, over email, and through website availability. In phase 1, 793 surveys were collected (46 from online responses and 747 from physical surveys). The second phase was interrupted by the 2020 COVID-19 pandemic and the original survey collection plans were adjusted. Surveys were collected from April 2020 to October 2020 through several methods, including using convenience sampling with email and website availability through social media. An address-based sample from census tracts with high percentages of AI/AN people was purchased through the Marketing System Group. Potential respondents were mailed a postcard with a link to an online survey. The final method of collection was through a telephone survey using a call center with the University of Oklahoma Health Sciences Center, the Sooner Survey Center. Phone numbers were also purchased through the Marketing System Group and the Sooner Call Center. Sixty-one surveys were collected through email and web availability, 23 were collected through the postcard mailing, and 295 were collected through the telephone collection survey. For evaluation purposes, to eliminate the effect of the Covid-19 pandemic, we used only the Phase I Oklahoma state sample data in this paper.

The initial target population of the BRFSS for Oklahoma state is all adults in Oklahoma. This target population is different from that of the TBRFSS, for which the target population of interest is Oklahoma American Indian adults. However, since the BRFSS used a probability sampling design (dual-frame random digit dialing method) for data collection, if we restrict the BRFSS data file to only Oklahoma American Indian adults, it is still a probability sample for that population, see Sect. 2.12 of the well-cited “Sampling techniques” book by Cochran (2007) [47]. Therefore, after restriction to Oklahoma American Indian adults, the two surveys have the same target population of interest.

## Methods

To combine the information from the 2018 and 2019 BRFSS, we stacked the two data files (e.g., 2018 and 2019 BRFSS) together. Then, we created the composite weight variable, which equals the original weight variable in the 2018 BRFSS (or 2019 BRFSS) divided by 2 if the corresponding participants belong to the 2018 BRFSS (or 2019 BRFSS). We also treated year and original stratification variables together as the new stratification variable after combining them. Other researchers [48–50] provided detailed discussions on creating composite weights for combining information from multiple surveys.

To evaluate the selection bias of the TBRFSS, we first compared the BRFSS weighted descriptive statistics (e.g., frequency and percentage for categorical variables, mean and standard deviation for continuous variables) and TBRFSS unweighted descriptive statistics for demographic and general health variables: Age Group, Gender, Marital Status, Education Level, Employment Status, Income Level, Body Mass Index (BMI), and General Health Status. The Rao-Scott Chi-square test [51, 52] was used to compare the distribution difference of categorical variables between the BRFSS and TBRFSS. Survey weighted regression [53] was used to compare differences in continuous variables between BRFSS and TBRFSS. Stratification and survey weights were incorporated.

The following data integration approaches were performed. First, the calibration approach was used, creating survey weights for TBRFSS by benchmarking the weighted descriptive statistics for variables (Age Group, Gender, Marital Status, Education Level, Employment Status, Income Level, BMI Status, and General Health Status) in the TBRFSS with those in the BRFSS. In other words, the calibrated weights ${\tilde{w}}_{i}$ for unit $i\in {s}_{B}$ where ${s}_{B}$ denotes the non-probability sample can be obtained by minimizing the distance function $\sum _{i\in {s}_{B}}\frac{1}{2}{\left(\frac{{\tilde{w}}_{i}}{{w}_{i}}-1\right)}^{2}$ such that $\sum _{i\in {s}_{B}}{\tilde{w}}_{i}{x}_{i}=\sum _{i\in {s}_{A}}{d}_{i}{x}_{i}$ where ${x}_{i}$ is a vector of covariate variables described above, ${w}_{i}$ is the initial weight in the non-probability sample (in our application, we used ${w}_{i}=1$), ${s}_{A}$ denotes the probability sample, and ${d}_{i}$ is the design weight in the probability sample for unit $i$. Specifically, an iterative proportional fitting algorithm [54, 55] was used to obtain the above calibration weights. Second, the sequential mass imputation approach by FCS method was used [43, 44]. Variables described in the calibration approach as well as the two-way interaction terms were used as the predictors in the sequential mass imputation model. Logistic regression models were used for categorical study variables. Linear regression models were used for continuous study variables. For both data integration approaches, we considered the following nine outcome variables of interest: Smoking status, Arthritis status, Cardiovascular Disease status (CVD), Chronic Obstructive Pulmonary Disease status (COPD), Asthma status, Cancer status, Stroke status, Diabetes status, and Health Coverage status. Even though the nine outcome variables were observed in both the BRFSS and TBRFSS data files, for evaluation purposes, we assumed that they were only observed in the TBRFSS for conducting the two data integration approaches. For the sequential mass imputation approach, we first used a non-probability sample (e.g., TBRFSS) to fit the imputation model, then generated imputed values of the above nine outcome variables sequentially once for the entire combined BRFSS data file. For simplicity, we only generated one imputed data file. Besides the assumption that there are overlapping covariate variables in both probability sample and non-probability sample, the validity of calibration approach depends on the assumption that there is a reasonable linear correlation between the outcome variables and covariate variables at the population level. The validity of mass imputation approach depends on the assumption that the imputation model fitted by using non-probability sample is reasonable and holds at the population level. If the associations between the covariate variables and outcome variables were small, then the data integration methods may not be very effective for reducing the selection bias. After data integration, we compared the adjusted estimates after data integration with the true estimates observed in the BRFSS. The Rao-Scott Chi-square test [51, 52] has been used to compare the distribution differences in categorical variables between different methods. Missing values for the above eight covariate variables and night outcome variables in both BRFSS and TBRFSS were imputed by using the SAS procedure ‘PROC MI’ before data integration. Since the missing rates for all variables are less than 5%, we only used single imputation. All analyses were conducted using SAS 9.4.

## Results

After subsetting to only the Oklahoma AI/AN adults, there were 635 observations in the 2019 TBRFSS and 973 observations in the 2018 and 2019 BRFSS combined data file. Table 1 presents the comparison between the BRFSS weighted descriptive statistics and the TBRFSS unweighted descriptive statistics for the variables listed in the Methods section. All variables were highly significant with p values less than 0.001. The TBRFSS sample tended to be older (45.04% vs. 27.42% for 55 plus), female (77.95% vs. 51.20%), have fewer married cases (38.11% vs. 44.31%), have higher education level (60.00% vs. 47.98% for above high school graduation), have higher employment status (62.99% vs. 57.80%), have lower income (9.92% vs. 20.73% for above $75,000), have higher BMI (55.75% vs. 40.30% for obesity), and have less general health (31.50% vs. 42.72% for Very Good and Excellent).

**Table 1:** Comparison of BRFSS weighted descriptive statistics, TBRFSS unweighted descriptive statistics, and TBRFSS weighted descriptive statistics after calibration process (Significant results with p values less than 0.001 are marked with *)

Variable	Value	BRFSS weighted Frequency (Percent)	TBRFSS original Frequency (Percent)	TBRFSS weighted Frequency (Percent)
age*	**18–24**	46,597 (17.07)	37 (5.83)	46,799 (17.15)
	**25–29**	30,027 (11.00)	48 (7.56)	30,027 (11.00)
	**30–34**	32,567 (11.93)	46 (7.24)	32,413 (11.88)
	**35–39**	29,459 (10.79)	49 (7.72)	29,365 (10.76)
	**40–44**	19,838 (7.27)	55 (8.66)	19,351 (7.09)
	**45–49**	17,961 (6.58)	51 (8.03)	17,759 (6.51)
	**50–54**	21,637 (7.93)	63 (9.92)	21,637 (7.93)
	**55–59**	21,303 (7.81)	94 (14.80)	22,338 (8.18)
	**60–64**	16,142 (5.91)	76 (11.97)	16,503 (6.05)
	**65–79**	12,267 (4.49)	59 (9.29)	11,967 (4.38)
	**70+**	25,129 (9.21)	57 (8.98)	24,768 (9.07)
gender*	**Male**	133,198 (48.80)	140 (22.05)	133,198 (48.80)
	**Female**	139,728 (51.20)	495 (77.95)	139,728 (51.20)
marital*	**Married**	120,946 (44.31)	242 (38.11)	120,875 (44.29)
	**Divorced/Separated**	50,397 (18.47)	142 (22.36)	50,397 (18.47)
	**Widowed**	16,701 (6.12)	60 (9.45)	16,772 (6.15)
	**Never Married**	72,022 (26.39)	114 (17.95)	72,022 (26.39)
	**Member of unmarried Couple**	12,861 (4.71)	77 (12.13)	12,861 (4.71)
education*	**Less than High School**	38,116 (13.97)	63 (9.92)	38,116 (13.97)
	**High School Graduate**	103,878 (38.06)	191 (30.08)	103,878(38.06)
	**Some college/technical school**	89,158 (32.67)	231 (36.38)	89,158 (32.67)
	**College Graduate**	41,774 (15.31)	150 (23.62)	41,774 (15.31)
employ*	**Employed/Self-employed**	157,742 (57.80)	400 (62.99)	157,742 (57.80)
	**Unemployed/Homemaker/Student**	49,507 (18.14)	72 (11.34)	49,507 (18.14)
	**Retired**	31,124 (11.40)	104 (16.38)	31,124 (11.40)
	**Unable to Work**	34,553 (12.66)	59 (9.29)	34,553 (12.66)
income*	**Less than $10,000**	24,554 (9.00)	117 (18.43)	20,620 (7.56)
	**Less than $15,000**	11,586 (4.25)	60 (9.45)	13,248 (4.85)
	**Less than $20,000**	32,404 (11.87)	63 (9.92)	33,385 (12.23)
	**Less than $25,000**	29,114 (10.67)	76 (11.97)	31,622 (11.59)
	**Less than $35,000**	35,740 (13.10)	88 (13.86)	37,572 (13.77)
	**Less than $50,000**	42,416 (15.54)	89 (14.02)	38,790 (14.21)
	**Less than $75,000**	40,524 (14.85)	79 (12.44)	41,062 (15.04)
	**$75,000 or More**	56,587 (20.73)	63 (9.92)	56,628 (20.75)
BMI Cat*	**Underweight/Healthy weight**	64,439 (23.61)	105 (16.54)	64,439 (23.61)
	**Overweight**	98,507 (36.09)	176 (27.72)	98,507 (36.09)
	**Obese**	109,980 (40.30)	354 (55.75)	109,980 (40.30)
general health*	**Excellent**	37,839 (13.86)	56 (8.82)	37,839 (13.86)
	**Very Good**	78,767 (28.86)	144 (22.68)	78,559 (28.78)
	**Good**	85,727 (31.41)	261 (41.10)	86,200 (31.58)
	**Fair/Poor**	70,593 (25.87)	174 (27.40)	70,329 (25.77)

Table 1 also presents the comparison between the BRFSS weighted descriptive statistics and the TBRFSS weighted descriptive statistics for the variables listed in the Methods section after the calibration process. As expected, the difference between the two distributions was small after calibration, which shows the improvement of the representativeness for the TBRFSS after calibration. For example, the weighted percentage of Male by using TBRFSS becomes 48.80% after calibration adjustment and it equals to the BRFSS weighted percentage. The weighted percentage of Married people by using TBRFSS becomes 44.29% after calibration adjustment and it is close to the BRFSS weighted percentage of 44.31%.

**Fig. 1:**
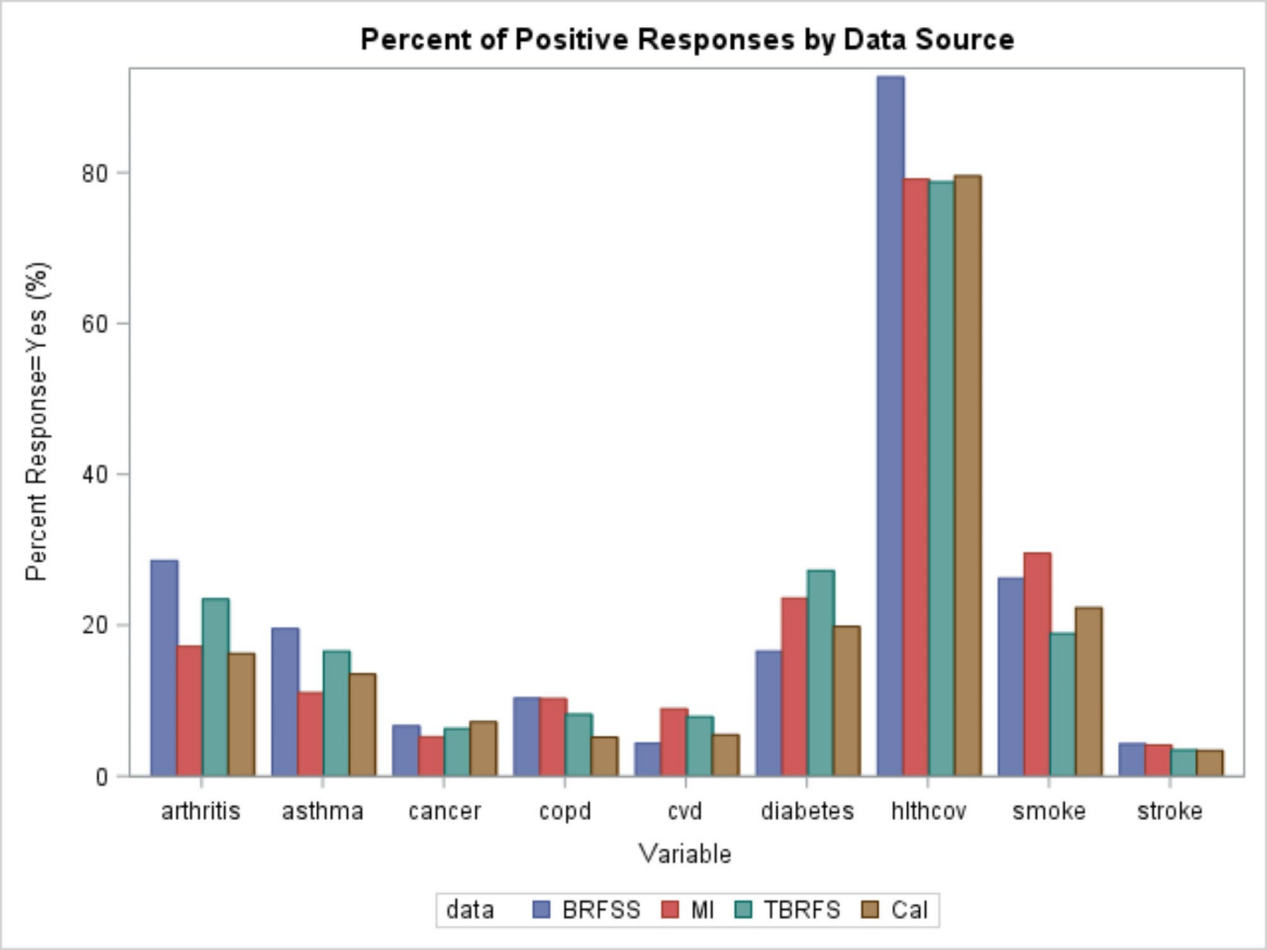
Comparison of data integration methods with nine outcome variables

Table 2; Fig. 1 present the comparison of two data integration methods (calibration and mass imputation) with the naïve method without any adjustment. In Table 2, the frequency (%) for mass imputation was computed by using the composite weight variable and the imputed outcome variables in the combined BRFSS data file. The frequency (%) for the calibration method was calculated based on the calibration weight for TBRFSS data. According to Table 2, both calibration and mass imputation significantly improved the estimates of frequency, since their estimates were much closer to the true estimates from the BRFSS (p values less than 0.001). The naïve estimates were significantly smaller than the true estimates in terms of frequency (p values less than 0.001). For percentages, data integration methods outperformed the naïve method for most variables, except Arthritis status and Asthma status. For example, the estimates of percentage of smoking status were 29.54% and 22.31% for the mass imputation and calibration methods, respectively, and they were closer to the true estimate from the BRFSS (26.22%) than the naïve estimate (18.90%) from the TBRFSS. The estimates of percentage of Diabetes status were 23.58% and 19.83% for the mass imputation and calibration methods, respectively, and they were closer to the true estimate from the BRFSS (16.54%) than the naïve estimate (27.24%) from the TBRFSS. The mass imputation method was significantly better at estimating the prevalence of smoking than the naïve estimate (p value less than 0.05). The calibration method was significantly better at estimating the prevalence of diabetes than the naïve estimate (p value less than 0.05). In general, the two data integration methods outperform naïve method by only using TBRFSS since they took advantage of the associations between covariate variables and outcome variables and also the representativeness of the probability sample.

**Table 2:** Comparison of data integration methods with nine outcome variables (Significant results with p values less than 0.001 are marked with *)

Variable	Value	BRFSS weighted	TBRFSS original	Mass imputation	Calibration
		Frequency (Percent)	Frequency (Percent)	Frequency (Percent)	Frequency (Percent)
**smoke**	**Yes**	71,571 (26.22)	120 (18.90)	80,619* (29.54)*	60,895* (22.31)
	**No**	201,354 (73.78)	515 (81.10)	192,306* (70.46)*	212,031* (77.69)
**arthritis**	**Yes**	77,919 (28.55)	149 (23.46)*	46,883* (17.18)	44,322* (16.24)
	**No**	195,007 (71.45)	486 (76.54)*	226,042* (82.82)	228,604* (83.76)
**cvd**	**Yes**	11,852 (4.34)	50 (7.87)	24,342* (8.92)	14,968* (5.48)
	**No**	261,073 (95.66)	585 (92.13)	248,583* (91.08)	257,958* (94.52)
**copd**	**Yes**	28,198 (10.33)	52 (8.19)	28,037* (10.27)	14,090* (5.16)
	**No**	244,727 (89.67)	583 (91.81)	244,888* (89.73)	258,835* (94.84)
**asthma**	**Yes**	53,322 (19.54)	105 (16.54)	30,155* (11.05)	36,904* (13.52)
	**No**	219,604 (80.46)	530 (83.46)	242,770* (88.95)	236,022* (86.48)
**cancer**	**Yes**	18,171 (6.66)	40 (6.30)	14,190* (5.20)	19,628* (7.19)
	**No**	254,755 (93.34)	595 (93.70)	258,736* (94.80)	253,297* (92.81)
**stroke**	**Yes**	11,685 (4.28)	22 (3.46)	11,251* (4.12)	9264* (3.39)
	**No**	261,240 (95.72)	613 (96.54)	261,674* (95.88)	263,661* (96.61)
**diabetes**	**Yes**	45,144 (16.54)	173 (27.24)	64,367* (23.58)	54,123* (19.83)*
	**No**	227,781 (83.46)	462 (72.76)	208,558* (76.42)	218,802*(80.17)*
**health coverage**	**Yes**	252,860 (92.65)	500 (78.74)	215,899* (79.11)	217,074* (79.54)
	**No**	20,066 (7.35)	135 (21.26)	57,026* (20.89)	55,851* (20.46)

## Discussion

Non-probability samples (e.g., convenience samples) have been used frequently in public health research due to the cost-effectiveness, time efficiency, and convenience of implementation. For instance, one component in the initial stage of the Strong Heart Study was a survey with a non-probability sample of American Indian tribal members 35–74 years of age residing in the three study areas (the community mortality study) to determine cardiovascular disease mortality rates from 1984 to 1994. Another well-known example is the Framingham Heart Study, which collected a non-probability sample of 5,209 men and women between the ages of 30 and 62 from the town of Framingham, Massachusetts, who had not yet developed overt symptoms of cardiovascular disease or suffered a heart attack or stroke. Furthermore, most surveys for American Indian health research, such as the TBRFSS and the Cherokee Nation Health Survey, used non-probability samples. Statistical analysis based on a non-probability sample without proper adjustment may lead to biased results due to selection bias. Data integration has been regarded as one of the most effective ways to reduce the selection bias from non-probability samples.

In this paper, we proposed a novel sequential mass imputation approach and applied it together with the calibration approach to improve the representativeness of the TBRFSS. To the best of our knowledge, we are the first to implement data integration approaches to American Indian Health research. Data integration procedures, including calibration and sequential mass imputation methods, show promising results for improving the representativeness of TBRFSS. Our proposed methods can be naturally applied to other health studies that use non-probability samples, such as the Strong Heart Study, the Framingham Heart Study, and many others. If selection biases or measurement errors were consistent over a period of time, then the trend analysis might still be possible. In addition, we developed user-friendly computational resource for other public health researchers. Computational codes can be obtained by sending email to the first author of this paper. The approach had limitations. First, we evaluated only the proposed data integration methods by using the BRFSS and TBRFSS. Further evidence must be provided by using other data files. Second, we considered only the nine outcome variables discussed in previous sections as examples. It might be interesting to evaluate other outcome variables. Thirdly, we only considered parametric sequential mass imputation approaches in the study. Nonparametric and machine learning methods can be used to improve the robustness of the methods. Lastly, there might be some small degree of misclassification for race variables in both BRFSS and TBRFSS. We ignored such effect in our study.

## Conclusion

In this paper, we compared two of the most popular data integration approaches (calibration and mass imputation) for AI/AN health research by combining the information from the 2018 and 2019 BRFSS and the 2019 TBRFSS. Both data integration approaches improved the representativeness of the original TBRFSS sample for all outcome variables in terms of frequency estimates and for most outcome variables in terms of percentage estimates. In practice, it can happen that the unweighted estimate from non-probability sample is closer to the weighted estimate from probability sample compared to the adjusted estimate since we only observe one random sample and the performance of the data integration methods depend on the underlying model assumptions of outcome variables and covariate variables. The approach also had strengths. First, we are the first to apply data integration methods to improve the representativeness of AI/AN survey data. Second, we are the first to propose using sequential mass imputation for data integration. In future research, we will propose and apply machine learning-based data integration approaches, including regression tree, random forest, XGboosting, and Deep Learning, to further improve the representativeness of the TBRFSS, since those machine learning approaches can better model the complex non-linearity in the data file and may have better prediction power. We also plan to apply our proposed data integration approaches to combine information from future TBRFSS surveys with the BRFSS and American Community Surveys. One can first build an imputation model by using the TBRFSS and then impute outcome variables of interest in both the BRFSS and American Community Surveys.

## Data Availability

2018 and 2019 Behavioral Risk Factor Surveillance System (BRFSS) data files are publicly available at the website https://www.cdc.gov/brfss/annual_data/annual_data.htm. The request of 2019 Tribal Behavioral Risk Factor Surveillance System (TBRFSS) may be available from the Southern Plains Tribal Health Board deputy director Cuyler Snider (csnider@spthb.org) on reasonable request.

## References

[1] National Diabetes Statistics Report. (2020). Estimates of Diabetes and Its Burden in the United States. https://www.cdc.gov/diabetes/pdfs/data/statistics/national-diabetes-statistics-report.pdf

[2] CDC. 2020. Summary Health Statistics: National Health Interview Survey: 2018. Table A-15a. https://www.cdc.gov/nchs/nhis/shs/tables.htm

[3] U.S. Department of Health and Human Services. Tobacco, Use Among US. Racial/Ethnic Minority Groups—African Americans, American Indians and Alaska Natives, Asian Americans and Pacific Islanders, Hispanics: A Report of the Surgeon General. Atlanta: U.S. Department of Health and Human Services, Centers for Disease Control and Prevention, National Center for Chronic Disease Prevention and Health Promotion, Office on Smoking and Health, 1998 [accessed 2018 Jun 12].

[4] Garrett BE, Dube SR, Winder C, Caraballo RS (2013). Cigarette Smoking—United States, 2006–2008 and 2009–2010. Morb Mortal Wkly Rep.

[5] Bureau USC. 2020 [cited 2021 03/07/2021]. Available from: https://data.census.gov/cedsci/table?q=american%20indian&tid=ACSDT1Y2019.B02010&hidePreview=false.

[6] Wiley Z, Kubes JN, Cobb J, Jacob JT, Franks N, Plantinga L et al. Age, Comorbid Conditions, and Racial Disparities in COVID-19 Outcomes. J Racial Ethn Health Disparities. 2021. Epub 2021 Jan 9. doi: 10.1007/s40615-020-00934-0. PubMed PMID: 33415702; PMCID: PMC7790329. 10.1007/s40615-020-00934-0 PMC7790329 33415702

[7] Wegermann K, Wilder JM, Parish A, Niedzwiecki D, Gellad ZF, Muir AJ (2021).

[8] McKnight-Eily LR, Okoro CA, Strine TW, Verlenden J, Hollis ND, Njai R (2021). Racial and ethnic disparities in the prevalence of stress and worry, Mental Health Conditions, and increased substance use among adults during the COVID-19 pandemic - United States, April and May 2020. MMWR Morb Mortal Wkly Rep.

[9] Krishnamoorthy G, Arsene C, Jena N, Mogulla SM, Coakley R, Khine J et al. Racial disparities in COVID-19 hospitalizations do not lead to disparities in outcomes. Public Health. 2021;190:93 – 8. Epub 2021 Jan 2. doi: 10.1016/j.puhe.2020.11.021. PubMed PMID: 33385640; PMCID: PMC7698674. 10.1016/j.puhe.2020.11.021 PMC7698674 33385640

[10] Purnell TS, Simpson DC, Callender CO, Boulware LE (2021). Dismantling structural racism as a Root cause of racial disparities in COVID-19 and transplantation. Am J Transplant.

[11] Nowotny KM, Bailey Z, Brinkley-Rubinstein L (2021). The contribution of prisons and jails to US racial disparities during COVID-19. Am J Public Health.

[12] Kim D, Lee Y, Thorsness R, Nguyen KH, Swaminathan S, Rivera-Hernandez M (2021). Racial and ethnic disparities in excess deaths among persons with kidney failure during the COVID-19 pandemic, March-July 2020. Am J Kidney Dis.

[13] Khatri UG, Pizzicato LN, Viner K, Bobyock E, Sun M, Meisel ZF (2021). Racial/Ethnic disparities in Unintentional Fatal and Nonfatal Emergency Medical Services-Attended opioid overdoses during the COVID-19 pandemic in Philadelphia. JAMA Netw Open.

[14] Escobar GJ, Adams AS, Liu VX, Soltesz L, Chen YI, Parodi SM (2021). Racial disparities in COVID-19 testing and outcomes: Retrospective Cohort Study in an Integrated Health System. Ann Intern Med.

[15] Clay SL, Woodson MJ, Mazurek K, Antonio B, Racial Disparities, Factors P. Health Access/Affordability, and Conditions Associated with an increased severity of COVID-19. Race Soc Probl. 2021;1–13. 10.1007/s12552-021-09320-9. Epub 2021 Feb 23. 10.1007/s12552-021-09320-9 PMC7880209 33613785

[16] Adepoju OE, Ojinnaka CO. County-Level Determinants of COVID-19 Testing and Cases: Are there Racial/Ethnic Disparities in Texas? Popul Health Manag. 2021. Epub 2021 Feb 6. doi: 10.1089/pop.2020.0300. PubMed PMID: 33544028. 10.1089/pop.2020.0300 33544028

[17] Gross CP, Essien UR, Pasha S, Gross JR, Wang SY, Nunez-Smith M (2020). Racial and ethnic disparities in Population-Level Covid-19 mortality. J Gen Intern Med.

[18] Polyakova M, Udalova V, Kocks G, Genadek K, Finlay K, Finkelstein AN (2021). Racial disparities in excess all-cause Mortality during the early COVID-19 pandemic varied substantially Across States. Health Aff (Millwood).

[19] Lopez L, Hart LH, Katz MH (2021). Racial and ethnic health disparities related to COVID-19. JAMA.

[20] Gold JA, Rossen LM, Ahmad FB, Sutton P, Li Z, Salvatore PP (2020). Race, ethnicity, and age trends in persons who died from COVID-19—United States, May–August 2020. Morb Mortal Wkly Rep.

[21] Hathaway ED, American Indian and Alaska Native People (2021). Social vulnerability and COVID-19. J Rural Health.

[22] John-Henderson NA, Mueller CM (2020). The relationship between health mindsets and health protective behaviors: an exploratory investigation in a convenience sample of american indian adults during the COVID-19 pandemic. PLoS ONE.

[23] Dyer O. Covid-19: Black people and other minorities are hardest hit in US. BMJ. 2020;369:m1483. Epub 2020 Apr 16. doi: 10.1136/bmj.m1483. PubMed PMID: 32291262. 10.1136/bmj.m1483 32291262

[24] Shekhar R, Sheikh AB, Upadhyay S, Atencio J, Kapuria D (2020). Early experience with COVID-19 patients at academic hospital in Southwestern United States. Infect Dis.

[25] Rodriguez-Lonebear D, Barceló NE, Akee R, Carroll SR (2020). Research full report: american indian reservations and COVID-19: correlates of early infection rates in the pandemic. J Public Health Manage Pract.

[26] Reyhner JA. Who and What Are American Indians? Race in America: How a Pseudoscientific Concept Shaped Human Interaction [2 volumes]. 2017:181.

[27] Crepelle A, Murtazashvili I. COVID-19, Indian Reservations, and Self-Determination. Mercatus COVID-19 Response Policy Brief. 2020.

[28] Kovich H (2020). Rural matters—coronavirus and the navajo nation. N Engl J Med.

[29] Solis J, Franco-Paredes C, Henao-Martínez AF, Krsak M, Zimmer SM (2020). Structural vulnerability in the US revealed in three waves of COVID-19. Am J Trop Med Hyg.

[30] Tyra AT, Ginty AT, John-Henderson NA (2021).

[31] John-Henderson NA, Ginty AT (2020). Historical trauma and social support as predictors of psychological stress responses in american indian adults during the COVID-19 pandemic. J Psychosom Res.

[32] Evans-Campbell T (2008). Historical trauma in american Indian/Native Alaska communities: a multilevel framework for exploring impacts on individuals, families, and communities. J interpers Violence.

[33] English KC, Espinoza J, Pete D, Tjemsland A (2019). A comparative analysis of telephone and in-person survey administration for public health surveillance in rural american indian communities. J Public Health Manage Pract.

[34] Watanabe-Galloway S, Duran T, Stimpson JP, Smith C (2013). Gaps in Survey Data on Cancer in American Indian and Alaska native populations: examination of US Population surveys, 1960–2010. Prev Chronic Dis.

[35] Baker R, Brick JM, Bates NA, Battaglia M, Couper MP, Dever JA (2013). Report of the AAPOR Task Force on non-probability sampling. J Surv Stat Methodol.

[36] Elliott MR, Valliant R (2017). Inference for nonprobability samples. Stat Sci.

[37] Yang S, Kim JK (2020). Statistical Data Integration in Survey Sampling: a review. Japanese J Stat Data Sci.

[38] Kim JK, Park S, Chen Y, Wu C. Combining Non-probability and Probability Survey Samples Through Mass Imputation.Journal of the Royal Statistical Society: Series A. 2021.

[39] Valliant R (2020). Comparing Alternatives for Estimation from Nonprobability samples. J Surv Stat Methodol.

[40] Chen JKT, Valliant R, Elliott MR. Surv Methodol. 2018;44:117–44. Model-Assisted Calibration of Non-Probability Sample Survey Data Using Adaptive Lasso.

[41] Yang S, Kim JK, Hwang Y. Integration of survey data and big observational data for finite population inference using mass imputation.Survey Methodology. 2021.

[42] Chen S, Yang S, Kim JK. Nonparametric Mass Imputation for Data Integration.Journal of Survey Statistics and Methodology. 2020. 10.1093/jssam/smaa036 PMC8784012 35083356

[43] Brand JPL. Development, Implementation, and Evaluation of Multiple Imputation Strategies for the Statistical Analysis of Incomplete Data Sets. Ph.D. thesis, Erasmus University. 1999.

[44] Van Buuren S (2007). Multiple imputation of discrete and continuous data by fully conditional specification. Stat Methods Med Res.

[45] Murray JS (2018). Multiple imputation: a review of practical and theoretical findings. Stat Sci.

[46] Burgette LF, Reiter JP (2010). Multiple imputation for missing data via sequential regression trees. Am J Epidemiol.

[47] Cochran WG. Sampling techniques. John Wiley & Sons; 2007.

[48] Chu A, Brick JM, Kalton G. Weights forcombining surveys across time or space. Bulletin of the International StatisticalInstitute: 52nd Session, ContributedPapers, Book 2. 1999: 103-4.

[49] Friedman EM, Jang D, Williams VT. Combined Estimates from FourQuarterly Survey Data Sets. 2002 Proceedings from the Joint StatisticalMeetings – Section on Survey ResearchMethods. 2002: 1064-69.

[50] Homas S, Wannell B (2009). Combining cycles of the Canadian Community Health Survey. Health Rep.

[51] Rao JNK, Scott AJ. Chi-Squared Tests for Goodness of Fit and Independence in Two-Way Tables. J Am Stat Assoc. 1981;76:221–30. The Analysis of Categorical Data from Complex Surveys:.

[52] Rao JNK, Scott AJ (1984). On Chi-Squared tests for Multiway Contingency tables with Cell Properties estimated from Survey Data. Ann Stat.

[53] Heeringa S, West BT, Berglund PA. Applied survey data analysis. Boca Raton, FL:Chapman & Hall. 2010.

[54] Kolenikov S (2014). Calibrating Survey Data using Iterative Proportional Fitting (Raking). Stata J.

[55] Valliant R, Dever JA, Kreuter F (2013). Practical tools for Designing and Weighting Survey samples.

